# Acne Vulgaris—Novel Treatment Options and Factors Affecting Therapy Adherence: A Narrative Review

**DOI:** 10.3390/jcm11247535

**Published:** 2022-12-19

**Authors:** Aleksandra Tobiasz, Danuta Nowicka, Jacek C. Szepietowski

**Affiliations:** Department of Dermatology, Venereology and Allergology, Wrocław Medical University, Chałubińskiego 1, 50-368 Wrocław, Poland

**Keywords:** acne vulgaris, dermatology, treatment, compliance, persistence

## Abstract

Acne vulgaris is an extremely common skin condition, affecting a large population of adolescents, but at the same time, remaining a quite common issue in the group of adult patients. Its complex pathogenesis includes increased sebum secretion, impaired follicular keratinization, colonization of sebaceous glands with *Cutibacterium acne* bacteria, and the development of inflammation in pilosebaceous units. Although there are many methods of treatment available targeting the mechanisms mentioned above, a large percentage of patients remain undertreated or non-compliant with treatment. Ineffective treatment results in the formation of acne scars, which has a major impact on the well-being and quality of life of the patients. The aim of this publication was a review of available evidence on widely used and novel methods of topical and systemic treatment of acne, additionally including current literature-based analysis of factors affecting patients’ compliance. The strengths and limitations of novel substances for treating acne were discussed. We conclude that an effective acne treatment remains a challenge. A better understanding of current treatment options and factors affecting patients’ compliance could be a helpful tool in choosing a proper treatment option.

## 1. Introduction

Acne vulgaris, very common in adolescence, is a condition of complex pathogenesis with a wide variety of treatment options with different mechanisms of action [1,2,3]. Because of its long duration and exposure to affected areas, acne is associated with a major deterioration in a patient’s quality of life and well-being [4,5,6]. According to the European evidence-based (S3) guideline for the treatment of acne [7], certain topical and systemic treatment options remain a standard. Nevertheless, there is still a need for novel treatment options. According to the guidelines, the intensity of treatment depends on the severity of the acne and should start with topical agents only—the combination of adapalene and benzyl peroxide or clindamycin with benzyl peroxide. In severe cases, it is recommended to begin treatment with systemic isotretinoin. Long-term therapy is essential for achieving certain therapeutic goals. Therefore, patient cooperation and adherence to doctor recommendations are of great importance. Since the largest group of patients struggling with acne are adolescents, achieving good cooperation can be a challenge [8].

## 2. Topical Treatment

Topical treatment of acne is widely used and effective, especially in cases of mild and moderate acne [7]. Various agents are commonly used, such as topical retinoids, antibiotics, benzoyl peroxide, azelaic acid, and salicylic acid [9]. Even though some of them are used in monotherapy, the advantage of various combinations of the above-mentioned substances in therapy has shown superior effects in many studies and is included in European guidelines [7,10,11]. What is more, many research studies indicate that agents such as topical antibiotics should not be used in monotherapy because of growing bacterial resistance and the limited action of such treatment [12,13].

In recent years, novel promising drugs have been introduced in topical form. Furthermore, medications used in dermatology to treat inflammatory disorders have attracted the attention of researchers due to their possible usefulness in the treatment of acne.

### 2.1. Retinoids

Topical retinoids have been used in the treatment of acne for more than 50 years, with all-trans retinoic acid (tretinoin, ATRA) being their first natural representative [14]. Activation of retinoic receptors by retinoids (RARα, RARβ and RARγ) results in gene transcription, which affects the growth and differentiation of skin cells. As a result, a comedolytic effect is reached, which is desired in the treatment of acne [15]. Over the years, new synthetic retinoids such as tazarotene and adapalene have been introduced in the treatment of acne [15,16,17]. In the last decade, the FDA has approved a new fourth-generation retinoid, trifarotene [18], which is a selective retinoic receptor gamma agonist characterized by better tolerability. It was initially approved for treating lamellar ichthyosis and later for treating acne vulgaris [19]. Many studies have proven its effectiveness and a favorable safety profile. In the study by Aubert et al. [20], its high pharmacological potency was confirmed in the pluristratified RHE model. In addition, in vivo, it eliminated almost all comedones using ten times lower dosages than used in the case of tazarotene and ATRA (classical retinoid-responsive rhino mouse model). The study by Tan et al. [21] was designed as a vehicle-controlled, double-blind, randomized, phase III study of 50 μg/g trifarotene cream once-daily vs. vehicle. This study lasted 12 weeks and included 1208 subjects with moderate facial and truncal acne. Trifarotene proved to be effective and safe with manageable tolerability. Another multicenter study by Blume-Peytavi et al. [22] and post-hoc analysis of two large-scale phase III pivotal trials by Eichenfield et al. [23] also confirmed the effectiveness, safety and tolerability of trifarotene. Not only are new synthetic retinol derivatives of great interest, but so are novel delivery systems. In recent years, various delivery systems of topical retinoids have been introduced, including polymeric nanoparticles, solid lipid nanoparticles, nanostructured lipid carriers, flexible liposomes, nanoemulsions, and microemulsions [24,25,26,27].

Microsponges are microscopic spheres with a diameter of 5–300 μm, which contain up to 250,000 pores. Therefore, they have specific properties; they slowly release the substance that is encapsulated in them, avoiding the accumulation of excessive amounts of the drug. The microsponge particles are too large to be absorbed through the skin, which increases their safety [28], resulting in a reduction of side effects characteristic of topical medication, such as skin irritation. Microsponge technology was patented in 1987 [29]. Regarding acne therapy, some products using this technology and containing retinoids have been introduced [30]. Numerous studies have shown that microsponge formulations allow the release of active substances in a controlled manner [31]. Two double-blind, randomized, split-face studies compared 0.1% tazarotene gel once daily versus 0.1% tretinoin microsponge gel once daily for the treatment of facial acne vulgaris. Tazarotene showed higher efficacy and similar tolerability, which made this medication a cost-effective alternative to 0.1% tretinoin microsponge gel [32,33]. Other studies focused on the properties of benzyl peroxide microsponge formulations [34,35]. Microsponges are definitely a very interesting and novel technology which could be used more frequently in the future.

### 2.2. Clascoterone

Clascoterone is a novel topical agent approved by the FDA in 2020 for the treatment of acne in patients 12 years of age and older [36]. It is a monoester of cortexolone (cortexolone 17alpha-propionate) with topical antiandrogenic activity. First, studies on rats indicated its apparent absence of systemic effects [37]. The study by Mazzetti et al. [38] investigated clascoterone cream in various concentrations (0.1%, 0.5%, and 1%) and confirmed its favorable safety and tolerability profile. In this study, the 1% cream proved to be the most effective, and it was selected for further clinical studies and development. Another study of the 1% cream in a group of patients with acne vulgaris showed the safety and tolerability of such a treatment [39]. Finally, a study by Hebert et al. [40] on the effects of treatment with 1% clascoterone cream in a group of 1440 patients with facial acne proved its efficacy and safety with low rates of adverse.

### 2.3. Dapsone

Another drug widely used in dermatology that has promising effects in the treatment of acne is dapsone. It is known to have a broad spectrum of action, including inhibition of neutrophil and eosinophil myeloperoxidase, inhibition of neutrophil adhesion to vascular endothelium integrins, inhibition of chemotaxis and generation of 5-lipogenase products in neutrophils and macrophages [15]. A 5% gel was approved by the FDA for the treatment of acne in 2005, and later, in 2016, its higher concentration of 7.5% also received such approval [41]. A 5% gel should be used twice a day [42], whereas a 7.5% gel is effective, safe, and well tolerated with once-a-day use [43]. Interestingly, a posthoc analysis of two clinical trials conducted by Tanghetti et al. [44] on the effect of 7.5% dapsone gel used for 12 weeks by patients with facial acne reported superior efficacy of such treatment in a female group and similar tolerability in male and female groups. Furthermore, a study by Taylor et al. [44] revealed the effectiveness of 7.5% dapsone gel in patients of all skin phototypes and good tolerability and safety of use. Finally, a study by Grove et al. [45] aimed to compare tolerability and irritation during topical use of benzyl peroxide 5%-clindamycin phosphate 1.2% versus benzyl peroxide 2.5%-clindamycin phosphate versus dapsone 5% and benzyl peroxide 2.5%-adapalene 0.1% in a group of healthy subjects indicated good tolerability of all the preparations mentioned above with higher frequency of adverse perceptions in the group of patients which used benzylperoxide 2.5%-adapalene 0.1%. Taking all studies into consideration, dapsone is a highly effective and well-tolerated form of topical treatment for acne vulgaris.

### 2.4. Calcipotriol

Calcipotriol, an analog of calcitriol, is widely used in the topical treatment of psoriasis in combination with betamethasone. Its mechanism of action involves binding to the vitamin D receptor of the nuclei of keratinocytes and suppression of keratinocyte proliferation [46]. A study by Abdel-Wahab et al. [47] performed between December 2021 and February 2022 investigated its use among 40 patients with mild and moderate acne vulgaris. In this study, patients were treated with 0.005% calcipotriol cream on the right side of the face and with 0.1% adapalene gel on the left side of the face once a day for two months. After two months of treatment, a significant reduction in comedones, inflammatory lesions, and total acne lesions was observed on each side of the face, with no statistically significant difference between the sides of the face. The analysis of skin biopsy showed greater anti-inflammatory potential of calcipotriol in comparison with adapalene. Additionally, calcipotriol was better tolerated by the patients than adapalene. The results of this study, although conducted in a small group of patients, are promising, proposing calcipotriol as a noteworthy form of topical treatment of acne.

### 2.5. Photodynamic Therapy

Photodynamic therapy uses the energy of visible light and a photosensitive drug such as aminolaevulinic acid, which is converted to protoporphyrin [48,49]. This process results in the production of 1O2, which has a highly reactive cytocidal action. It has been mostly used in the treatment of actinic keratosis, basal cell skin cancer, cutaneous T-cell lymphoma, etc. [50,51,52,53].

Many recent studies showed its effectiveness in the treatment of severe acne vulgaris. According to Yang et al. [54], photodynamic therapy with 5% ALA-PDT and red light is effective in the treatment of acne conglobate with a high response rate and reduced scar formation. A study by Liu et al. [55] investigating a combination of 5-aminolevulinic acid photodynamic therapy and isotretinoin in a group of 67 patients showed its effectiveness in the treatment of moderate to severe acne.

Pain seems to be the most bothering side effect [56], while other side effects include edema, erythema, and hyperpigmentation [57]. A study by Wojewoda et al. [58] indicated that the use of methyl aminolevulinate (MAL-PDT), shorter incubation time, and smaller doses could increase the tolerability of treatment. Interestingly, Zhang et al. [59] noted that the use of 5% and 10% ALA-PDT on different sides of the face of patients with severe facial acne vulgaris resulted in a slightly higher pain level with 10% gel on the pain score, but there were no differences in experienced pain between used concentrations during the second, third and fourth session of photodynamic therapy.

There is a high need for a protocol with fixed concentrations of photosensitizing agents, light dose, number of sessions, and incubation time, which would combine the highest possible effectiveness with the least bothersome side effects.

## 3. Systemic Treatment

Although isotretinoin remains a golden standard for the systemic treatment of severe acne, because of its adverse effects [15] and teratogenic action [53], there is still a need for a systemic drug with a better safety profile and fewer side effects. According to European guidelines [7], another option for systemic treatment of severe acne is antibiotics in combination with topical adapalene or azelaic acid. The antibiotics of choice remain doxycycline and lymecycline but are limited to a treatment period of three months.

### 3.1. Sarecycline

The development of bacterial resistance during the treatment with systemic antibiotics has become a great concern. In recent years, a higher resistance of *Cutibacterium acnes* to antibiotics was described in various studies [60,61,62]. That is why novel antibiotics with safer action profiles are being developed. An example of such an antibiotic is sarecycline, a tetracycline-derived oral antibiotic, which was approved by the FDA in 2018 for the treatment of moderate to severe acne vulgaris [63]. It shows higher selectivity for *C. acnes* compared to older-generation tetracyclines because there is a lower risk of developing bacterial resistance during treatment [64]. According to clinical studies, sarecycline has shown good effectiveness in the treatment of severe facial and truncal acne, with a relatively low rate of side effects and good tolerability [65,66,67].

### 3.2. Montelukast

An interesting acne therapeutic option described in various studies is montelukast. Montelukast is a selective CysLT1 receptor antagonist with an anti-inflammatory effect of action [68]. In Poland, this drug is registered for the treatment of asthma [69]. A 2015 study by Behrangi et al. [70] in a group of 52 patients with moderate acne comparing the treatment with oral doxycycline 100mg/day plus topical 1% clindamycin and with montelukast 5mg plus 1% topical clindamycin reported a significant reduction in the acne severity index in both groups, without significant differences between the groups. Another study by Rokni et al. [71] in a group of 65 women with moderate acne vulgaris evaluating treatment with the combination of oral montelukast and finasteride with topical clindamycin showed good efficacy of both treatment methods with the advantage of finasteride. Whereas the study by Fazelzadeh-Haghighi et al. [72] in a group of 108 patients with moderate acne vulgaris comparing a treatment with doxycycline 100mg/day with montelukast 10mg/day and doxycycline 100 mg/day with placebo showed a superior effect of the montelukast/doxycycline combination versus doxycycline alone. Both groups also received a topical 5% benzyl peroxide gel to use once every night. Surely montelukast is an interesting agent, but its effectiveness as an adjuvant in the therapy of acne requires further studies.

### 3.3. Hormonal Therapy

Hormonal therapy for acne, particularly in women, is an important option because women after 25 years of age suffer from frequent relapses of acne following standard treatment. There are several hormones that can contribute to the development of acne, including androgens. Although in those women, symptoms of acne appear even if androgens remain within the normal range. This group of patients shows good results after hormonal treatment [73].

## 4. Adherence

An issue without which successful therapy is impossible is the compliance of the patients. Furthermore, Cramer et al. [74] indicate that for treatment to be successful, not only is compliance (adherence) key, but persistence is also of great importance. In this study, after three years of review and discussion by the ISPOR Work Group, the following definitions were proposed according to which medication compliance “… refers to the act of conforming to the recommendations made by the provider with respect to timing, dosage, and frequency of medication taking.” And medication persistence “… refers to the act of conforming to a recommendation of continuing treatment for the prescribed length of time.”

Considering the long duration of acne treatment, the visibility of the lesions, and the struggle with various side effects, these two concepts are especially important. A large observational study on the adherence to acne therapy by Dreno et al. [75] on more than 3000 patients from all over the world using multivariate analysis indicated eight factors that had predictive power in relationship to adherence (two historical factors, three clinical factors, and three related to the current treatment). It permitted the establishment of a patient profile more likely to be poorly adherent to acne treatment. Historical factors were the severity of the disease and previous consultation with a general practitioner about acne. Clinical factors included patients’ age, satisfaction with treatment, and knowledge about acne treatment. Patients with poor adherence were more often school-aged, single, with symptom onset before puberty, and with lower levels of knowledge about acne. Characteristics of the current treatment regarded the additional anti-acne cosmetics prescription, the degree of clinical improvement evaluated by the physician, and the occurrence of side effects. The study resulted in the Elaboration d’un outil d’evaluation de l’observance des traitements medicamenteux (ESOB) questionnaire, which can help doctors to identify patients with poor adherence. Another study by Hayran et al. [76] included a group of 500 patients and reported poor treatment adherence to treatment in 64.4% of the patients. Identified factors related to better adherence to treatment were the use of oral isotretinoin and satisfaction with treatment.

Comparing adherence between topical and systemic acne vulgaris treatment results vary between available studies. A study by Hayran et al. [76] mentions better adherence of patients using oral isotretinoin (83.7% of patients). Interestingly the study by Salamzadeh et al. [77], which investigated adherence in a group of 200 patients with mild, moderate, and severe acne vulgaris in Iran, indicated no significant difference between adherence to topical versus systemic treatment. On the other hand, studies by Dreno et al. [75] as well as Miyachi et al. [78] indicated higher adherence to topical treatment. Even though mentioned studies described general adherence to the treatment of patients with acne vulgaris as poor, a study by Alsubeeh et al. [79] included a group of 2330 patients with psoriasis, chronic dermatitis, acne vulgaris, hair growth disorders, and vitiligo, indicated better adherence of patients with acne vulgaris than the ones with psoriasis or chronic dermatitis. The duration of the treatment might be a possible explanation. In the study of Yentzer et al. [80], in the group of teenagers using 5% gel with benzoyl peroxide, a large decline in adherence during six weeks of treatment was described. Even such a short duration of treatment affected patients’ adherence.

Patients’ persistence, on the other hand, has fewer studies than adherence. Nevertheless, some interesting observations have been described. A study by Grada et al. [81] investigated persistence in a group of 230,552 patients with acne, and it indicated a relatively high Medication Possession Ratio (percentage of patients for whom medication is available) but poor treatment persistence.

Considering all the above-mentioned information, proper education of the patient is of great importance. Regarding choosing a proper medium study by Hung et al. [82] showed that a larger group of patients preferred educational videos over pamphlets. That is why educational videos are a useful tool in the education of patients about the treatment. 

## 5. Conclusions

Acne is a multi-factorial skin disease that requires long-term treatment. Over the years, many topical and systemic treatment options have been introduced; however, many patients do not see satisfactory treatment results and experience difficulties adhering to treatment recommendations. Therefore, selecting the appropriate treatment is of great importance for achieving satisfactory treatment outcomes that match patient needs and ensure patient cooperation. 

## Data Availability

Not applicable.
